# Association of residual feed intake with growth and slaughtering performance, blood metabolism, and body composition in growing lambs

**DOI:** 10.1038/s41598-017-13042-7

**Published:** 2017-10-04

**Authors:** Xiaoxue Zhang, Weimin Wang, Futao Mo, Yongfu La, Chong Li, Fadi Li

**Affiliations:** 1 The State Key Laboratory of Grassland Agro-ecosystems, College of Pastoral Agriculture Science and Technology, Lanzhou University, Lanzhou, 730020 China; 2 College of Animal Science and Technology, Gansu Agricultural University, Lanzhou, 730000 China; 3 Engineering Laboratory of Sheep Breeding and Reproduction Biotechnology in Gansu Province, Minqin, 733300 China

## Abstract

The aim of this study was to determine the association of residual feed intake (RFI) with growth performance, blood metabolic parameters, and body composition factors in growing lambs. Individual body weight (BW) and dry matter intake (DMI) were determined in 137 male Hu lambs that were given a pellet feed four times a day for 50 d. RFI did not show a correlation with metabolic BW (MBW) or average daily gain (ADG), but it showed a positive correlation with DMI and feed conversation ratio (FCR). Organ weight and intestine length had a large influence on RFI in lambs. The low-RFI lambs have smaller rumen and longer duodenum indicating the less feed intake and more sufficient absorption rate of low-RFI lambs. The smaller organs like liver, lung and kidney in low-RFI lambs may be related to lower energy consumption and slower metabolic rate. The observed bigger testis was in low-RFI lambs was another cause of the improved feed efficiency. Finally, the plasma concentrations of thyroxine (T4) and adrenocorticotropic hormone (ACTH) were lower in the ELow-RFI group than in the EHigh-RFI group. This study provides new insight into the biological processes underlying variations in feed efficiency in growing lambs.

## Introduction

Feed accounts for 65–70% of the cost in the sheep industry, and thus, improving feed efficiency (FE) is important for the economy and the environment. FE is a major indicator of the efficiency of feed utilization. When FE is low, there are negative consequences on the environment and the production cost is higher^1,2^. FE is represented by the feed conversation ratio (FCR) or residual feed intake (RFI). FCR is defined as the ratio of feed intake to weight gain over a specific period of time, and is traditionally used in meat and egg production; however, it has certain statistical and biological limitations^3–5^. Besides, measuring the FCR is not cost-effective. It was Koch *et al*. who proposed the use of RFI^6^, which is regarded as a sensitive and accurate method to estimate FE^7–9^. RFI is defined as the difference between the actual feed intake and the predicted intake based on the body size and performance of each animal. A low RFI indicates less feed consumption and less waste generation with no effect on the weight, production and body size of the animals. Thus, RFI may be a reliable indicator of the differences in FE that account for the diverse genetic background of animals. Further, studying the regulation mechanism of RFI can not only reduce the cost of feed but also protect the environment by reducing the emission of carbon and methane^10^.

Many factors affect RFI, including body composition, the digestion and metabolism of nutrients, energy output, body activity and body temperature regulation^11,12^. Most studies focus on pigs, cattle and poultry, and studies on sheep are few. The aim of this study was to determine the associations between RFI, slaughtering performance, blood metabolic parameters and body composition in growing sheep.

## Results

### Growth performance and feed efficiency

In this study, the mean DMI of the animals was 1.22 kg/d (SD = 0.18); ADG, 0.25 kg/d (SD = 0.03); and FCR, 4.90 kg of DMI/kg of BW gain (SD = 0.59). The mean RFI was 0.00 kg/d (SD = 0.09) and ranged from −0.31 to 0.22 kg/d, which represents a difference of 0.53 kg of feed per day between the animals with the highest and lowest RFI. The intake, growth performance, and FE data are presented in Table 1.

**Table 1:** Characterization of intake and growth performance in lambs with high, medium, and low residual feed intake (RFI).

**Trait**	**RFI group** ^1^			**SE** ^2^	***P*** **-value**
	**Low**	**Medium**	**High**		
No. of animals	42	54	41		
RFI, kg/d	−0.10^c^	0.00^b^	0.11^a^	0.05	< 0.0001
Feed conversion ratio, kg of DM/kg of BW gain	4.51^c^	4.84^b^	5.39^a^	0.01	<0.0001
DMI, kg/d	1.09^c^	1.25^b^	1.33^a^	0.02	<0.0001
Metabolic BW, kg^0.75^	12.97	13.18	13.16	0.11	0.724
ADG, kg/d	0.25	0.26	0.26	0.00	0.108
Initial BW, kg	24.44	24.76	24.94	0.34	0.933
Final BW, kg	36.66	37.66	37.34	0.39	0.935
Relative growth^3^, %	40.33	41.9	40.25	0.51	0.303

^a–c^Least square means within a row with different superscripts differ significantly (*P* < 0.05). ^1^High = RFI was > 0.5 SD above the mean; Medium = RFI was 0.5 SD above and below the mean; Low = RFI was < 0.5 SD below the mean. ^2^SE = pooled SE. ^3^Relative growth = 2(W_2_ − W_1_)/(W_1_ + W_2_) *100%, W_1_ = Initial BW, W_2_ = Final BW.

Low-RFI lambs consumed 12.8% and 18.0% less feed than their medium- and high-RFI counterparts, respectively (*P* < 0.001). The least-square means for RFI and FCR in the high-RFI lambs were higher than those in the the medium-RFI lambs (*P* < 0.001), while the least-square means for the medium-RFI lambs were greater than those for the low-RFI lambs (*P* < 0.001). There was no significant difference in ADG, initial BW, MBW, and final BW (*P* > 0.05) between the high-, medium-, and low-RFI groups. There was no significant difference in the relative growth rate either.

**Figure 1:**
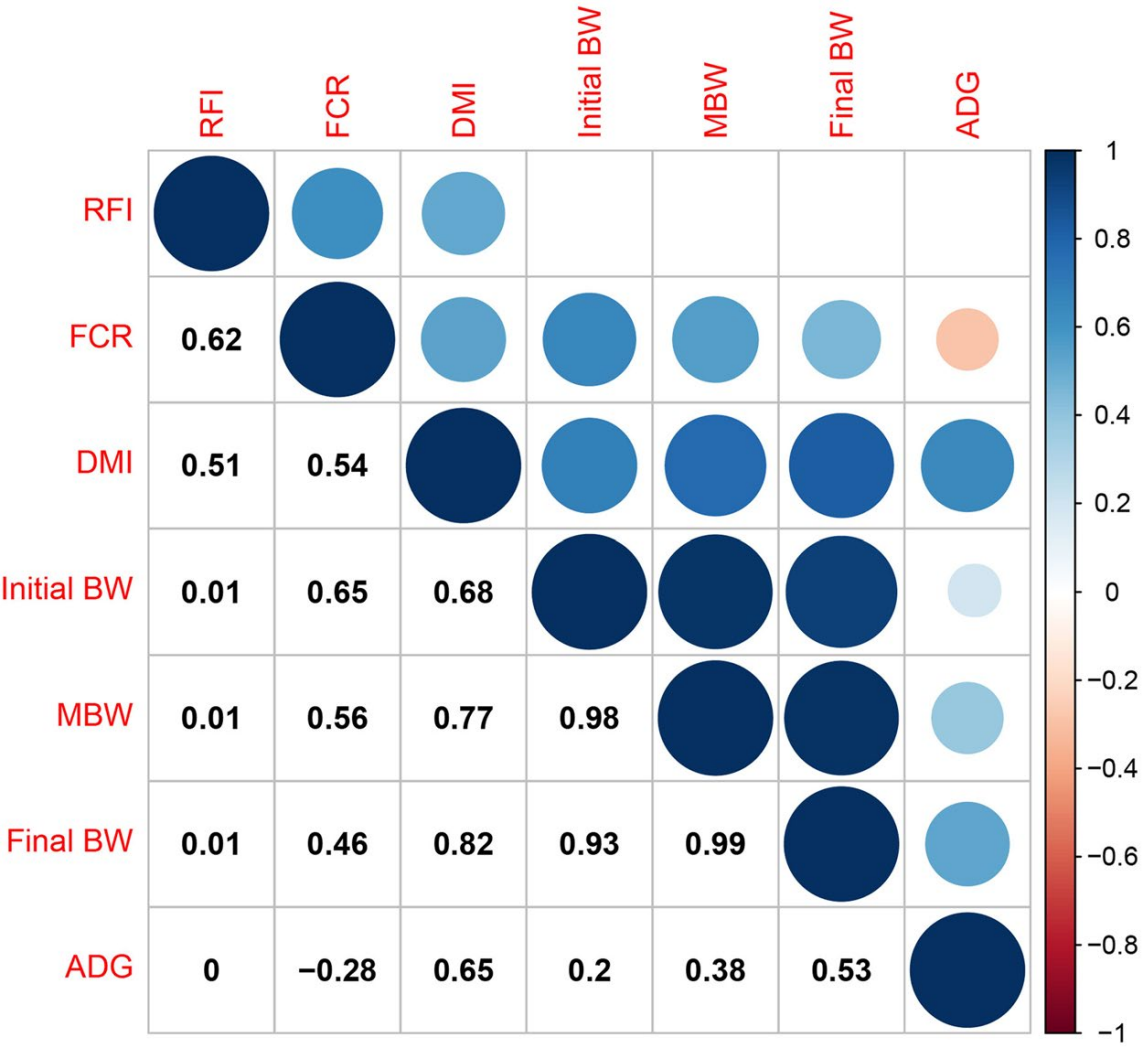
Correlation coefficient between intake, performance, and feed efficiency traits. Each circle represents the correlation coefficient between any two traits. The size of the circular indicates the absolute value of the correlation coefficient. Blue and red colour gradients indicate a positive or negative in correlation coefficient, respectively.

RFI was not significantly correlated with MBW, ADG, initial BW or final BW, but it was highly significantly correlated (*P* < 0.001) with DMI (r = 0.51) and FCR (r = 0.62). Further, DMI showed a highly significant correlation (*P* < 0.001) with ADG (r = 0.65), initial BW (r = 0.68), final BW (r = 0.82) and MBW (r = 0.77), and moderate correlation (*P* < 0.001) with FCR (r = 0.54). FCR showed a negative correlation with ADG (*P* < 0.01) (Fig. 1).

### Carcass traits

**Figure 2:**
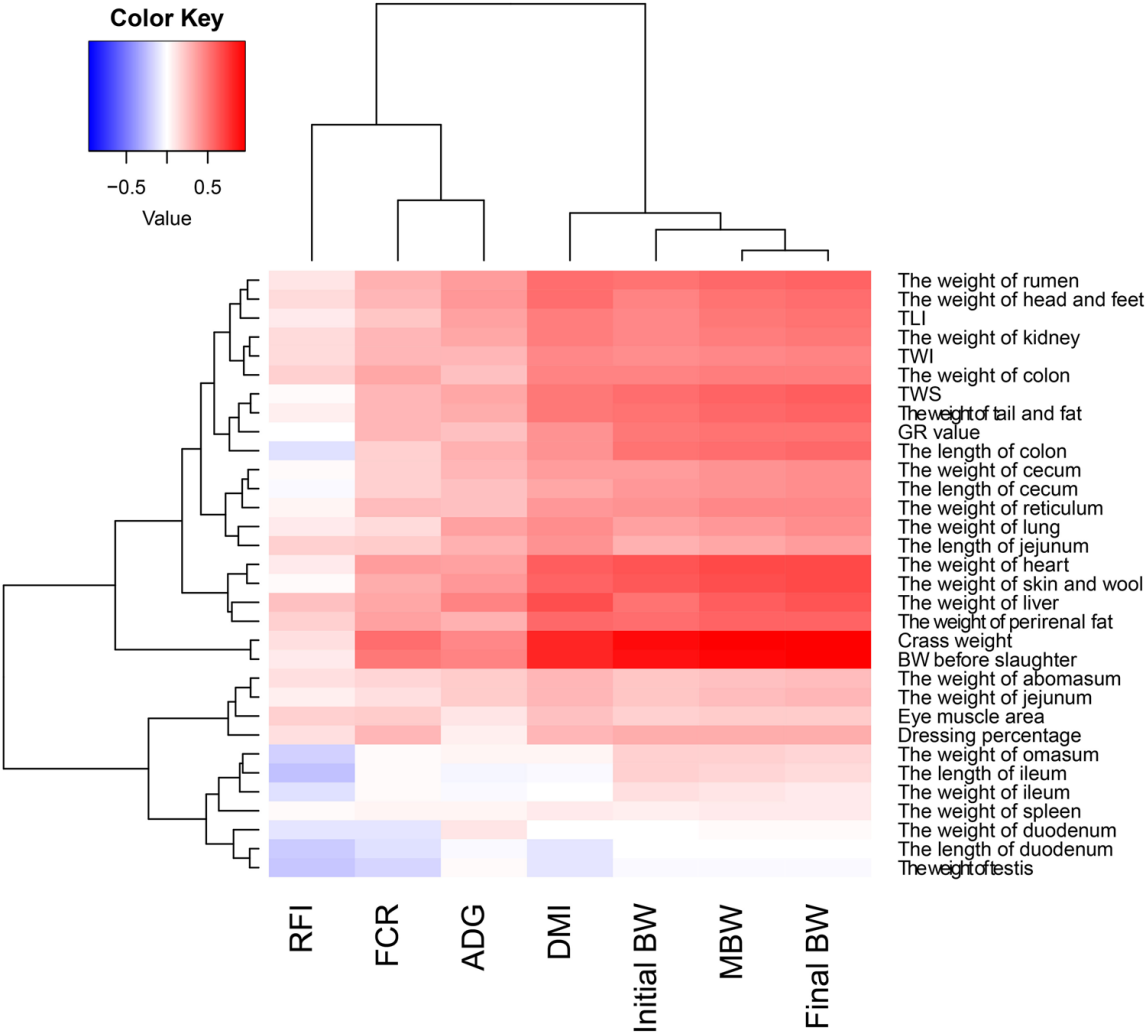
Coefficients of correlation between the intake, performance, FE traits and body composition. Data are shown in a analysis generated using the R software. Blue and red colour gradients indicate a negative or positive in correlation coefficient, respectively.

Differences in carcass traits between the three RFI groups are presented in Table 2. There was no significant difference (*P* > 0.05) between the RFI groups with regard to carcass traits, but a positive association was found between carcass traits, DMI, ADG, MBW, FCR, and RFI (*P* < 0.05) (Fig. 2). The association of DMI with each of the carcass traits was positive (*P* < 0.05). Further, the association of ADG with body weight, carcass weight, GR value, and tail fat weight was positive (*P* < 0.05), but ADG was not significantly correlated with RFI (*P* > 0.05). The correlation between FCR, body weight, crass weight, and GR value was positive. Moreover, RFI was positively associated with the eye muscle area and back fat thickness (*P* < 0.05).

**Table 2:** Characterization of carcass traits in lambs with low, medium, and high residual feed intake (RFI).

**Traits**	**RFI group** ^1^			**SEM** ^2^	***P*** **-value**
	**Low**	**Medium**	**High**		
No. of animals	42	54	41		
BW before slaughter, kg	39.27	41.09	39.70	0.34	0.063
Carcass weight, kg	19.01	19.88	19.68	0.18	0.175
GR value	16.65	17.89	17.11	0.23	0.117
Tail fat weight, kg	0.98	0.99	0.90	0.03	0.903
Eye muscle area, cm^2^	21.20	21.49	22.58	0.30	0.194
Back fat thickness, cm	0.79^b^	0.79^b^	0.95^a^	0.03	0.054
Dressing percentage, %	48.12	48.19	48.71	0.13	0.082

^a and b^Least-square means within a row with different superscripts differ significantly (*P* < 0.05). ^1^High = RFI was > 0.5 SD above the mean; Medium = RFI was 0.5 SD above and below the mean; Low = RFI was < 0.5 SD below the mean. ^2^SE = pooled SE.

### Tissue and visceral organs

The weight and percentage of tissue and visceral organs are presented in Table 3. The weight of the liver and lung were lower in low-RFI lambs than in medium-RFI and high-RFI lambs (*P* < 0.01), but the percentage weight of these two organs was not significantly different between the RFI groups. The weight and percentage weight of the testes were higher in the low-RFI and medium-RFI lambs than in the high-RFI lambs (*P* < 0.01). The total weight of the stomach and the total intestinal weight in the low-RFI lambs were 5.28% (*P* < 0.01) and 5.27% (*P* < 0.01) lesser than those in the high-RFI lambs. The results of correlation analysis indicated that RFI showed a positive correlation with the weight of liver and a negative correlation with the weight of testis (*P* < 0.01) (Fig. 2).

**Table 3:** Characterization of tissues and organs in lambs with high, medium, and low residual feed intake (RFI).

**Items**		RFI group^1^			SEM^2^	*P*-value
		**Low**	**Medium**	**High**		
No. of animals		42	54	41		
Heart	Weight, g	158.75	160.25	161.55	2.17	0.883
	Percentage, %	0.40	0.39	0.40	0.00	0.186
Liver	Weight, g	633.31^b^	694.12^a^	696.39^a^	8.87	0.005
	Percentage, %	1.62^b^	1.68^ab^	1.73ª	0.02	0.056
Spleen	Weight, g	51.40	52.71	52.62	0.66	0.681
	Percentage, %	0.13	0.15	0.13	0.00	0.547
Lung	Weight, g	375.87^b^	405. 41^ab^	408.04^a^	5.27	0.022
	Percentage, %	0.98	1.02	0.98	0.01	0.192
Kidney	Weight, g	110.12^a^	116.35^b^	114.74^b^	0.82	0.056
	Percentage, %	0.28	0.28	0.28	0.00	0.067
Perirenal fat	Weight, g	191.67	210.43	218.77	6.61	0.263
	Percentage, %	0.50	0.52	0.55	0.02	0.392
Head & feet	Weight, g	2.95	3.01	3.05	0.03	0.315
	Percentage, %	7.57	7.34	7.63	0.06	0.120
Skin & wool	Weight, g	4.70	4.84	4.76	0.06	0.655
	Percentage, %	11.99	11.69	11.83	0.11	0.517
Testis	Weight, g	234.10^a^	210.92^a^	158.43^b^	8.15	0.001
	Percentage, %	0.60^a^	0.51^b^	0.40^b^	0.02	0.001
TWS^3^	Weight, g	900.25^b^	956.52ª	950.51^a^	7.85	0.004
	Percentage, %	2.28	2.34	2.32	0.01	0.246
TWI^4^	Weight, g	1188.71^b^	1254.63^a^	1254.87^a^	9.90	0.007
	Percentage, %	3.06	3.16	3.16	0.02	0.331

^a–c^Least square means within a row with different superscripts differ significantly (*P* < 0.05). ^1^High = RFI was > 0.5 SD above the mean; Medium = RFI was 0.5 SD above and below the mean; Low = RFI was < 0.5 SD below the mean. ^2^SE = pooled SE. ^3^TWS = total weight of the stomach. ^4^TWI = total weight of the intestinal tract.

### Gastrointestinal tract

The gastrointestinal tract data for the high-, medium-, and low-RFI groups are presented in Table 4. Rumen weight was greater in medium- and high-RFI lambs than in low-RFI lambs (*P* < 0.01). Further, the reticulum weight in low-RFI animals was lesser than that in medium-RFI animals (*P* < 0.05), but it was not significantly different from that of the high-RFI animals (*P* > 0.05). The weight of the jejunum and colon in low-RFI animals was less than that in the medium- and high-RFI animals (*P* < 0.05).

**Table 4:** The weight of the gastrointestinal tract in lambs with high, medium, and low residual feed intake (RFI).

**Items**		RFI group^1^			SEM^2^	*P*-value
		**Low**	**Medium**	**High**		
No. of animals		42	54	41		
Rumen	Absolute weight, g	546.39^b^	587.49^a^	587.02^a^	5.09	0.001
	Relative weight, %	62.26	61.51	61.20	0.31	0.455
Reticulum	Absolute weight, g	93.75^b^	102.81^a^	98.86^b^	1.29	0.041
	Relative weight, %	10.47	10.70	10.58	0.15	0.798
Omasum	Absolute weight, g	108.06	117.33	112.40	1.81	0.137
	Relative weight, %	11.93	12.24	11.92	0.17	0.665
Abomasum	Absolute weight, g	144.94	149.52	152.59	2.16	0.427
	Relative weight, %	16.00	15.55	16.31	0.24	0.321
Duodenum	Absolute weight, g	37.39	37.54	36.19	0.75	0.736
	Relative weight, %	3.18	2.93	2.88	0.06	0.110
Jejunum	Absolute weight, g	764.01^b^	811.72^a^	785.63^ab^	7.63	0.031
	Relative weight, %	63.8	62.72	62.30	0.35	0.199
Ileum	Absolute weight, g	25.24	24.59	24.29	0.50	0.748
	Relative weight, %	2.11	1.91	1.94	0.04	0.108
Cecum	Absolute weight, g	47.32	52.37	49.21	0.88	0.052
	Relative weight, %	3.97	4.02	3.90	0.05	0.666
Colon	Absolute weight, g	320.39^b^	372.38^a^	369.41^a^	7.23	0.005
	Relative weight, %	26.94^b^	28.41^b^	28.97^a^	0.35	0.060

^a–c^Least square means within a row with different superscripts differ significantly (*P* < 0.05). ^1^High = RFI was > 0.5 SD above the mean; medium = RFI was 0.5 SD above and below the mean; low = RFI was < 0.5 SD below the mean. ^2^SE = pooled SE.

With regard to the intestinal length, the absolute and relative length of the duodenum and ileum in the low-RFI animals was greater than that in the medium- and high-RFI animals, respectively (*P* < 0.05). However, the length of the cecum was the shortest in the low-RFI lambs (*P* < 0.05). No significant differences were observed with regard to the other parts of the gastrointestinal tract (Table 5).

**Table 5:** Intestinal length in lambs with high, medium, and low residual feed intake (RFI).

**Items**		RFI group^1^			SEM^2^	*P*-value
		**Low**	**Medium**	**High**		
No. of animals		42	54	41		
Duodenum	Absolute length, cm	75.04^a^	61.47^b^	66.31^b^	1.71	0.004
	Relative length, %	2.25^a^	1.82^b^	1.92^b^	0.05	0.001
Jejunum	Absolute length, m	25.39^b^	26.01^ab^	26.53^a^	0.19	0.064
	Relative length, %	75.87^b^	76.71^ab^	77.15ª	0.22	0.079
Ileum	Absolute length, cm	36.68^a^	32.98^ab^	31.92^b^	0.80	0.048
	Relative length, %	1.10^a^	0.97^b^	0.93^b^	0.02	0.010
Cecum	Absolute length, cm	32.53^b^	35.91^a^	33.23^ab^	0.61	0.045
	Relative length, %	0.98	1.06	0.97	0.02	0.057
Colon	Absolute length, m	6.64	6.61	6.56	0.09	0.942
	Relative length, %	19.83	19.45	19.03	0.21	0.337
Total intestine	Total length^3^, m	33.46	33.92	34.40	0.23	0.290

^a–c^Least square means within a row with different superscripts differ significantly (*P* < 0.05). ^1^High = RFI was > 0.5 SD above the mean; medium = RFI was 0.5 SD above and below the mean; low = RFI was < 0.5 SD below the mean. ^2^SE = pooled SE. ^3^Total length represents the total length of the intestinal tract.

The results of correlation analysis showed that the weight of rumen was positive correlated with RFI, FCR, ADG, DMI, initial BW, MBW, and final BW (*P* < 0.05). The length and weight of duodenum were negative correlation with RFI (*P* < 0.01) and FCR (*P* < 0.05) (Fig. 2).

### Blood hormones and metabolites

Metabolic hormone and metabolite data for the EHigh-RFI and ELow-RFI animals are presented in Table 6. Correlation coefficients for the association of intake, performance, and FE traits with the metabolic variables are presented in Table 7. The plasma concentrations of T4 and ATCH were lower in the ELow-RFI group than in the EHigh-RFI group (*P* < 0.01). However, the concentrations of insulin, leptin, IGF-1, GC, and TRH were not significantly different between the groups (*P* > 0.05). RFI showed a positive correlation with T4 (R^2^ = 0.435, *P* < 0.05) and ACTH (R^2^ = 0.534; *P* < 0.01). FCR was positively correlated with T4 (R^2^ = 0.413, *P* < 0.05), but no other significant associations were observed with regard to the remaining traits (*P* > 0.05).

**Table 6:** Characterization of metabolic hormones and metabolites in lambs with EHigh and ELow residual feed intake (RFI).

**Items**	**RFI group** ^**1**^		**SEM** ^**2**^	**P-value**
	**ELow**	**EHigh**		
No.	15	15		
T4^3^, ng/mL	57.28^b^	79.52ª	4.32	0.007
Insulin, μIU/mL	9.69	11.44	0.69	0.211
ACTH^4^, pg/mL	24.39^b^	34.77^a^	1.78	0.002
Leptin, ng/mL	7.98	7.97	0.13	0.977
IGF-1^5^, ng/mL	15.40	18.20	2.10	0.519
GC^6^, ng/mL	21.73	21.71	1.17	0.991
TRH^7^, ng/L	49.87	41.49	2.27	0.066

^a,b^Least square means within a row with different superscripts differ significantly (*P* < 0.05). ^1^The EHigh group comprised 15 lambs with the highest RFI; the ELow group comprised 15 lambs with the lowest RFI. ^2^SE = pooled SE. ^3^T4 = thyroxine. ^4^ACTH = adrenocorticotropic hormone. ^5^IGF-1 = insulin-like growth factor-1. ^6^GC = glucocorticoid. ^7^TRH = thyrotropin-releasing hormone.

**Table 7:** Correlation of intake, performance, and feed efficiency traits with metabolic hormones and metabolites across all animals.

**Items**	**DMI**	**ADG**	**MBW** ^1^	**FCR** ^**2**^	**RFI** ^**3**^
T4^3^, ng/mL	0.276	−0.085	0.034	0.413^*^	0.435^*^
Insulin, μIU/mL	−0.041	−0.034	−0.272	0.005	0.251
ACTH^4^, pg/mL	0.207	0.002	−0.201	0.275	0.534^**^
Leptin, ng/mL	0.025	−0.044	0.149	0.103	−0.066
IGF-1^5^, ng/mL	−0.013	−0.118	−0.074	0.102	0.144
GC^6^, ng/mL	−0.008	−0.160	0.062	0.152	0.022
TRH^7^, ng/L	−0.021	0.148	0.261	−0.171	−0.379

^1^MBW = metabolic BW. ^2^FCR = feed conversion ratio. ^3^RFI = residual feed intake. ^3^T4 = thyroxine. ^4^ACTH = adrenocorticotropic hormone. ^5^IGF-1 = insulin-like growth factor-1. ^6^GC = glucocorticoid. ^7^TRH = thyrotropin-releasing hormone. ^†*^ *P* < 0.05; ^**^ *P* < 0.01.

## Discussion

In the present study, the base RFI regression model (DMI explained by MBW and ADG) accounted for 80% of the variation in DMI; in the current study, this is similar to the data of other studies on cattle^13,14^. DMI, FCR and RFI in low-RFI lambs were significantly lower than those in medium- and high-RFI lambs, but there were no differences in the initial BW, final BW, ADG, MBW and relative growth rate between the three groups, which was in agreement with the findings of Faure *et al*.^15^. The results indicate that selection of RFI in sheep could increase FE by reducing feed consumption without affecting the growth performance of sheep. In agreement with our findings, Cai *et al*. and Barea *et al*. found that RFI was generally not correlated with ADG, but was correlated with DMI^16,17^. Lancaster *et al*. considered RFI to be moderately correlated with FCR^18^. However, Nkrumah observed that RFI was strongly correlated with FCR, and that high-RFI steers that were fed a concentrate-based diet consumed 15% more feed than low-RFI steers, which was generally consistent with our results^19^. Previous studies have revealed that FCR is negatively correlated with ADG in lambs^20^, which concurs with our findings that applying selection pressure over a long period for FCR results in an increase in growth rate and mature size and leads to greater maintenance energy costs and thus an increase in feed requirement^21^. Nevertheless, previous results and our conclusion both imply that RFI has no association with ADG, initial BW, and final BW. Because the inheritance of RFI is independent of weight and ADG, genetic improvements using RFI as an index for FE can eliminate the effect of growth on RFI. Therefore, RFI is an accurate and sensitive index for measuring FE.

Controversies over the relationship between RFI and carcass traits have existed over the last few years. Most research results show that RFI has a weakly positive phenotypic and genetic correlation with body fat content^14,22,23^. In contrast to these findings, Herd and Bishop reported that RFI and carcass lean content had a negative phenotypic (r = −0.22) and genetic (r = −0.43) association^24^. In our study, the correlation coefficient between RFI and back fat (BF) was 0.227, and the BF in low-RFI animals was lower than that in high-RFI animals. This finding indicates although the potential benefit of selection of low-RFI animals is the reduction of BF deposition, it may mean an increase in the lean content of the carcass^22,25^.

At present, there is no known association of RFI with the depth of the longissimus dorsi or growth in steers, bulls, and heifers^5,14,19^. This is in agreement with our results, which show that there are no differences in GR, relative growth and slaughter rate between the three RFI groups. Our conclusion thus supplements the findings of previous studies. However, RFI has been reported to be weakly positively correlated with the eye muscle area^18,26^. Our correlation data also revealed that RFI showed a positive correlation with the eye muscle area (r = 0.188) and BF (r = 0.227). Further, the association of RFI and DMI with the eye muscle area and BF indicates that sheep with a higher RFI have greater feed intake and better muscle development, as well as more fat deposition.

The weight of the internal organs and its contribution to the total body weight reflect the health condition in animals^27^. The size of visceral organs is related to the level of feed intake^28^, as the energy expenditure of these organs increases after feeding and is dependent on feed intake^29^. In agreement with the findings of Basarab *et al*.^7^, who observed that low-RFI cattle had an 8% and 10% lighter liver and lung, respectively, than high-RFI cattle, the weight of the liver and lung in our lamb population were different among the RFI groups: the basal metabolic rate and respiration rate in the high-RFI group were higher than those in the low-RFI group. Moreover, the weight of testis was higher in low-RFI lambs than that in medium- and high-RFI lambs, and there was a significant negative correlation between RFI, FCR and the weight of testis. In agreement with our findings, Heaton *et al*. found that the greater testis size has higher feed efficiency in cattle^30^. Interestingly, the weight of the rumen was lower in low-RFI lambs and it had a medium positive correlation with DMI, which may lead to less feed intake in low-RFI lambs. No difference was observed in the total length of the intestinal tract, but it was showed that the length of the duodenum and ileum in low-RFI lambs was longer than that in high-RFI lambs and both of them had a negative correlation with FCR and RFI. The variation can be explained as that the longer duodenum and ileum improved the nutrients absorption in low-RFI animals, which results in high FE.

The systemic concentrations of various metabolic and nutrient uptake variables and inhibitors of tissue catabolism have been found to be potential physiological biomarkers of FE in cattle^31–33^. Walker *et al*. reported that T4 was affected by BW but not by RFI during the lactation and post-weaning period in cows^34^. In the later growth stages, Walker *et al*. found that the plasma level of T4 was not affected by BW but by RFI; this difference may be due to the role of thyroid hormones, as the developing heifers required thyroid hormones to remain active^35^. The results of previous studies are consistent with our findings that the plasma concentration of T4 was lower in ELow-RFI lambs than in EHigh-RFI lambs; thus, thyroid hormones are active in growing lambs.

Body composition, metabolic rate, and stress are some factors that influence energy use in animals^21^. Animals increase their metabolic rate in response to stress, which means that energy consumption and utilization also increase and the function of the hypothalamic-pituitary-adrenal axis is subsequently altered^36^. Knott *et al*. reported that an animal’s serum cortisol response to exogenously administered ACTH is strongly correlated with FE (measured using RFI) in an unselected line of rams^37^. This report is in agreement with the findings reported in chickens divergently selected for RFI^38^. In this study, the plasma concentrations of ACTH were lower in ELow-RFI lambs than in EHigh-RFI lambs, as high concentrations of ACTH increase excitability and lead to the loss of large amounts of energy in the form of heat energy, finally resulting in a decrease in feed efficiency.

According to Stick *et al*. and Wood *et al*. (2004), the blood concentrations of IGF-1 are potential physiological markers of FE and are phenotypically positively correlated with RFI in beef cattle^32,39^. However, Kelly *et al*. reported significant negative correlations between RFI and IGF-1 receptors in heifers divergent for RFI^40^. Furthermore, Kelly *et al*. reported that the correlations between serum IGF-1 concentrations and RFI varied between different sampling times on the same day^41^. In the present study, the serum concentrations of IGF-1 did not differ between different RFI groups; this is in agreement with the study of Richardson *et al*.^42^. Thus, the environment has a bigger impact on the concentration of metabolites than genetic mechanisms. Welch *et al*. reported that the genetic relationship between plasma IGF-I concentration and RFI becomes less positive as cattle mature physiologically. Thus, the genes associated with systemic IGF-I concentration differ between the post-weaning and finishing stages of development^43^. As a consequence, the concentration of serum IGF-1 may not be an appropriate indicator of RFI in sheep.

Leptin is known to regulate BW, feed intake, energy expenditure^44^, reproduction^45^, and immunocompetence^46^. The plasma concentration of leptin is correlated with body lipid depots^47,48^. Richardson *et al*. observed that the serum leptin concentration had a significant phenotypic correlation (r = 0.31) with RFI. In contrast, Brown *et al*. reported that systemic leptin concentration was not associated with intake, performance, or FE traits^49^; this is similar to our findings that the serum concentrations of leptin were not significantly different between ELow-RFI animals and EHigh-RFI animals. The contradictory results may be explained by the differences in the environment, animal breed and physiological status.

Previous studies have reported that the systemic insulin concentration in high-RFI steers is greater than that in low-RFI steers; this is believed to result from the decrease in leanness caused by an increase in fat deposition, as insulin can reduce lipolysis and stimulate lipogenes in adipose tissue^31^. In contrast, Nascimento *et al*. found higher blood insulin concentrations in low-RFI animals^50^. In the present study, plasma insulin concentrations had no relationship with intake, performance, or FE in sheep; this was similar to the results of Kelly *et al*. that were reported in growing beef heifers^41^.

## Conclusions

The findings of the current study indicate that there are significant differences in performance and FE in growing lambs. We observed a 18% decrease in DMI between low and high RFI lambs, but no difference in growth performance was detected between RFI groups. Organ weight and intestine length had a large influence on RFI in lambs. The low-RFI lambs have smaller rumen and longer duodenum indicating the less feed intake and more sufficient absorption rate of low-RFI lambs. The smaller organs like liver, lung and kidney in low-RFI lambs may be related to lower energy consumption and slower metabolic rate. The observed bigger testis was in low-RFI lambs was another cause of the improved feed efficiency, but the underlying mechanism remains to be investigated. Some level of association was observed between physiological markers and FE, for example, between T4 and ACTH and RFI during the growing period in lambs. Our present data show that high RFI lambs have physiology differences from low RFI lambs that control intake and conversion, but since this study was limited to a single breed and specific environmental conditions only, more research on larger populations of different breeds in different environments would be useful.

## Materials and Methods

### Ethics Statement

All experiments in this study were carried out in accordance with the approved guidelines from the Regulation of the Standing Committee of Gansu People’s Congress. All experimental protocols and the collection of samples were approved by the Ethics Committee of Gansu Agriculture University.

### Animals and management

In total, 137 male Hu lambs were purchased from Jinchang Zhongtian Sheep Industry Co. Ltd., Gansu, China, and transferred to Minqin Zhongtian Sheep Farm, at 90 days of age. Healthy lambs with good growth and intact genealogical records were randomly selected and treated with a standardized immunization program before they were weaned. They were reared indoors and housed in individual pens (0.8 × 1 m) until 165 days of age. The feeding and housing conditions and the management environment were standardized.

Briefly, the animals were exposed to an acclimatization period of 15 days, during which the proportion of pellet feed in the diet was gradually increased by 6.7% per day while the forage proportion was concurrently reduced until they were only fed the pellet feed. During this adaptation period, the animals were allowed to accustom themselves to the pellet feed and ad libitum feeding. After that, the lambs were on ad libitum intake for 10 days (pre-test period) before the experimental period, which lasted for 50 days. The feed intake for each animal was recorded in the pretest period in order to customize the feed intake in the experimental period. The animals were also given ad libitum access to fresh drinking water. Each sheepfold was thoroughly disinfected twice a month. At the start of the performance test, the mean BW was 24.72 kg (SD = 3.95). Experimental rations were formulated according to the recommendations of the feeding standard for sheep in China (NY/T816-2004). The feeds were stored at −20 °C in triplicate before they were analyzed for dry matter (DM), crude protein (CP), neutral detergent fiber (NDF) and acid detergent fiber (ADF). The other feed parameters were calculated according to the feed composition and nutritive values reported in China (2015)^51^. In order to determine the DM, the feed was oven dried at 104 °C for a minimum period of 16 h. The CP (Total N × 6.25) was determined using a previously reported method (Sweeney, 1989) with a VELP UDK 192 nitrogen analyzer. The NDF and ADF concentrations of the feed were determined using developed by Van Soest *et al*.^52^. The ingredients and chemical composition of the pellet feed in the experiment are shown in Table 8. The diet was processed into granules; the granulating temperature was 70 °C and the grain diameter was 6 mm. The health condition of lambs was observed by the veterinarian routinely, the lamb did not suffer from acidosis and they were all in good condition during the study.

**Table 8:** Ingredients and chemical composition of the experimental diet (air-dry basis).

**Ingredient**	**Percentage (%)**	**Chemical composition** ^**2**^	**Content**
Barley straw	27.00	DM (%)	87.55
Corn	44.00	CP (%)	16.28
Soybean meal	2.20	DE (MJ/kg)	12.38
Rapeseed meal	2.60	NDF (%)	36.54
Cottonseed meal	4.20	ADF (%)	14.12
Concentrate feed^1^	20.00	Ca (%)	0.60
Total	100.00	P (%)	0.30
		Starch (%)	28.48

^1^Concentrate feed consists of dried malt root, Urea, NaHCO_3_, Premix (2.5%), and NaCl (2.5%)0. ^2^Premix provides the following mineral elements (mg/kg) and vitamins (IU/kg): S, 200; Fe, 25; Zn, 40; Cu, 8; Mn, 40; I, 0.3; Se, 0.2; Co, 0.1; VA, 940; VD, 111; VE, 20. DM, CP, NDF, and ADF are the measured values, while the others are calculated values.

### Feed intake and growth data

The lambs were fed four times a day, at 0630, 1130, 1530 and 1900 h, and the amount of feed consumed was recorded. An independent crib was assigned to each lamb. To avoid the accumulation of feed in crib, feed remaining in the crib in excess of 20% of the feed offered daily was replaced by fresh feed. The amount of residual feed was weighed every morning before feeding and the samples of residual feed were analyzed for DM. Feed intake was measured for each animal based on the feeding amount and residual feed amount. Average DMI was then calculated using the total DMI for 50 days. Lambs were weighed in the morning before feeding, and at 0, 10, 20, 40 and 50 days of the experimental recording period using calibrated electronic scales. Five measurements were taken per animal over the course of the experiment. Weight was measured on two consecutive days at the five time points mentioned above.

### Blood collection and analysis

At the end of the experimental period, blood samples for 30 lambs (15 lambs with the highest RFI and 15 lambs with the lowest RFI) were obtained by jugular venipuncture in the morning. Samples were collected in EP tubes to determine the plasma concentrations of thyroxine (T4), insulin, ACTH, leptin, IGF-1, glucocorticoid (GC) and thyrotropin-releasing hormone (TRH). The samples were immediately stored in ice water; at the time of analysis, they were centrifuged at 1,500 × *g* at 4 °C for 15 min. The plasma was then split and stored in a new EP tube at −20 °C until analysis. The concentration of plasma IGF-I was determined using a validated RIA, according to a previously reported method by Spicer *et al*.^53^. The plasma concentrations of leptin, insulin, T4, and ACTH were determined with commercial RIA kits (Beijing North Institute of Biological Technology, China), and the concentrations of GC and TRH were determined by ELISA^5^.

### Slaughtering measurements

Ten days after the experimental period finished, lambs were transported to a commercial slaughterhouse. Lambs were weighed after 24 h of fasting and slaughtered in a standardized procedure. All procedures were in accordance with the guidelines of the Biological Studies Animal Care and Use Committee, Gansu Province, P. R. of China. All lambs were bled to death with a clean small cut to the jugular vein. Internal organs were removed from the body, residual blood was allowed to drip out, and the organs were then weighed. Each carcass was weighed within 30 min after slaughtering, and the dressing percentage was calculated after determining the BW from the carcass weight. The longissimus dorsi (LD) excised from the left carcass side at the 12^th^ rib was used to determine the eye muscle area (EMA). EMA was measured by planimeter on traced outlines of a cross section of the eye of LD at the 12^th^ rib^54^. The fat thickness of the 12^th^ rib represents back fat thickness (BF)^54^. The GR value represents the fat content of the carcass, which is based on the soft tissue depth at the GR site^54^. The GR site is present over the 12^th^ rib, at 110 mm away from the midline. The gastrointestinal tract was separated and ligatured using a cotton thread. The weight of the gastrointestinal tract was measured after cleaning and eliminating the contents. The length of the intestinal tract was measured using a tape.

### Determination of RFI

The feed intake of each animal was recorded over 50 days, from 115 days of age to 165 days of age, and the data were used to calculate the RFI for each lamb. RFI is defined as the difference between the actual daily feed intake and the expected daily feed intake of each individual^6^. RFI was calculated using a linear regression model, into which the DMI, ADG and mid-test metabolic body weight (MBW) data of all the lambs were entered. Total daily DMI was considered as the sum of all the meals consumed in a day, after correction for DM content. In the test period, ADG was calculated as the coefficient of the linear regression of BW (kg) on time with the REG procedure (SAS Inst. Inc., Cary, NC). MBW was calculated using the methods of Basarab *et al*.^14^.

The base model used was Y_i_ = β_0_ +  β_1_MBW_i_ + β_2_ADG_i_ + e_i_, where Y_i_ represents the DMI of the i^th^ animal; β_0_, the regression intercept; β_1_, the regression coefficient on MBW; β_2_, the regression coefficient on ADG; and e_i_, the uncontrolled error of the i^th^ animal.

For analysis of growth performance, FE, carcass traits, tissue and visceral organs, and gastrointestinal tract, the animals were divided into three groups based on the RFI values: high RFI (RFI > 0.5 SD above the mean), medium RFI (RFI ± 0.5 SD above and below the mean), and low RFI (RFI < 0.5 SD below the mean).

For blood hormone and metabolite analysis, the 15 lambs with the highest RFI (called the Extreme-High-RFI group, or the EHigh-RFI group for short) and the 15 lambs with the lowest RFI (called the Extreme-Low-RFI group, or the ELow-RFI group for short) were selected from the 137 lambs.

### Statistical analysis

In the data analysis, lambs were used as the experimental units. Statistical analysis was performed using SPSS 16.0 for Windows (SPSS, Chicago, IL, USA). Differences in growth performance, FE, carcass traits, the weight of tissue and visceral organs, and the weight and length of the gastrointestinal tract between the high-, medium-, and low-RFI groups were analyzed using ANOVA and LSD post-hoc test. Differences in blood parameters between the EHigh-RFI and ELow-RFI groups were determined using a *t*-test. A *P* value of < 0.05 was considered to indicate statistical significance. Data are presented as the mean ± SE values. Pearson correlation coefficient was calculated using the PROC CORR procedure.
